# Expression and Purification along with Evaluation of Serological Response and Diagnostic Potential of Recombinant Sap2 Protein from *C. parapsilosis* for Use in Systemic Candidiasis

**DOI:** 10.3390/jof7120999

**Published:** 2021-11-23

**Authors:** Manisha Shukla, Pankaj Chandley, Harsimran Kaur, Anup K. Ghosh, Shivaprakash M. Rudramurthy, Soma Rohatgi

**Affiliations:** 1 Department of Biosciences and Bioengineering, Indian Institute of Technology Roorkee, Roorkee 247667, India; mshukla@bt.iitr.ac.in (M.S.); Pankaj_c@bt.iitr.ac.in (P.C.); 2 Department of Medical Microbiology, Postgraduate Institute of Medical Education and Research, Chandigarh 160012, India; drharsimranpgi@gmail.com (H.K.); anupkg3@gmail.com (A.K.G.); mrshivprakash@yahoo.com (S.M.R.)

**Keywords:** Sap2, *C. parapsilosis*, antibody, ELISA, systemic candidiasis, diagnosis

## Abstract

Systemic candidiasis is the fourth most common bloodstream infection in ICU patients worldwide. Although *C. albicans* is a predominant species causing systemic candidiasis, infections caused by non-albicans *Candida* (NAC) species are increasingly becoming more prevalent globally along with the emergence of drug resistance. The diagnosis of systemic candidiasis is difficult due to the absence of significant clinical symptoms in patients. We investigated the diagnostic potential of recombinant secreted aspartyl proteinase 2 (rSap2) from *C. parapsilosis* for the detection of *Candida* infection. The rSap2 protein was successfully cloned, expressed and purified using Ni-NTA chromatography under denaturing conditions using an *E. coli*-based prokaryotic expression system, and refolded using a multi-step dialysis procedure. Structural analysis by CD and FTIR spectroscopy revealed the refolded protein to be in its near native conformation. Immunogenicity analysis demonstrated the rSap2 protein to be highly immunogenic as evident from significantly high titers of Sap2-specific antibodies in antigen immunized Balb/c mice, compared to sham-immunized controls. The diagnostic potential of rSap2 protein was evaluated using immunoblotting and ELISA assays using proven candidiasis patient serum and controls. Immunoblotting results indicate that reactivity to rSap2 was specific to candidiasis patient sera with no cross reactivity observed in healthy controls. Increased levels of anti-Sap2-specific Ig, IgG and IgM antibodies were observed in candidiasis patients compared to controls and was similar in sensitivity obtained when whole *Candida* was used as coating antigen. In summary, the rSap2 protein from *C. parapsilosis* has the potential to be used in the diagnosis of systemic candidiasis, providing a rapid, convenient, accurate and cost-effective strategy.

## 1. Introduction

Fungal pathogens belonging to the genus *Candida* can cause various infections ranging from superficial mucocutaneous infections to devastating invasive candidiasis in humans [1]. Systemic candidiasis is one of the most common bloodstream infections in hospitalized patients worldwide [1,2] and is associated with a 40–70% mortality rate, even in the presence of antifungal therapies [3,4,5]. The increasing incidence of candidiasis worldwide over recent years, resulting in high morbidity and mortality, is a cause for concern. *Candida* species are ranked as the fourth most common cause of nosocomial bloodstream infections in the United States and seventh in Europe [6,7]. Amongst the approximately 20 medically relevant *Candida* species identified [8], *C. albicans* has been the predominant species isolated historically [9]. However, a change in the epidemiology towards non-albicans *Candida* (NAC) species has been observed over the past few decades [10,11]. The prevalence of non-albicans *Candida* (NAC) species is currently on the rise, collectively accounting for about 65% of *Candida* infections [12,13]. The most common non-albicans *Candida* species involved in the infectious processes are *C. tropicalis*, *C. parapsilosis*, *C. krusei*, *C. glabrata*, *C. guilliermondii*, *C. lusitaniae* and *C. haemulonii*, to name a few [14,15]. More recently, a multi-drug-resistant strain named *C. auris* has been identified [16,17]. Every year, systemic candidiasis affects approximately 250,000 people world-wide causing ~50,000 deaths. As per the Centre for Disease Control and Prevention (CDC), an estimated 34,000 cases of candidiasis were reported in hospitalized patients resulting in 1700 deaths in 2017 [18]. Recently, epidemiological studies in SARS-CoV-2 infections reported that secondary invasive candidiasis infections are associated with considerable morbidity and mortality [19].

To reduce the mortality associated with systemic candidiasis, early and correct identification is essential. Studies have shown that late and incorrect diagnosis of *Candida* species leads to a significant increase in mortality [20,21]. The identification of patients at risk of systemic candidiasis is complicated due to the usual lack of characteristic clinical manifestations, specific clinical signs and symptoms. Due to the absence of an effective early diagnosis method, definitive diagnosis of these conditions is often delayed, and their subsequent therapeutic outcomes are poor. Current diagnostic methods for candidiasis depend on traditional microbiological culture, which is relatively insensitive and time-consuming [22,23]. Moreover, this method often results in late diagnosis during an advanced stage of infection. This subsequently causes a delay in the antifungal treatment and significantly reduces the survival rate of candidiasis patients. Urinal or mucosal positive cultures can also be seen during systemic infections, but they do not necessarily detect invasive candidiasis. Further, the insensitivity of microbiologic cultures results in delayed diagnosis and definitive treatment, which increase mortality and medical costs [24]. Cultures of blood samples collected under sterile conditions have long been accepted as gold standards for diagnosis of invasive candidiasis [25] but blood cultures are limited in diagnosing invasive candidiasis by poor sensitivity and the slow turn-around time [1,5]. The role of blood cultures in clinical practice is quite limited due to the low sensitivity and positive results, which appear only in advanced stages of infection [26]. The problems associated with the microbiological culture method highlight the need to develop more reliable, rapid, sensitive and specific diagnostic tests.

Certain non-culture approaches have been developed and evaluated to overcome the limitations of the conventional microbiological culture method [22]. They involve the detection of either the fungal nucleic acids, metabolites, antigens or antibodies from candidiasis patients [27,28,29,30]. Various studies have demonstrated the use the (1,3)-β-d-glucan-based assay in the diagnosis of *Candida* infection [31,32]. However, it has been also reported that structural similarities of the cell surface antigen (1, 3)-β-d-glucan in various pathogens can give rise to false-positive results [33]. The *C. albicans* cell wall mannan antigen has also been used in the serological diagnosis of systemic candidiasis [30,34]. There are several available antibody-based *Candida* detection assays, which include *C. albicans* germ tube antibodies (CAGTA) and anti-mannan antibodies [30]. Although PCR-based diagnostic assays have been developed [35,36], commercially available *Candida* DNA-based detection techniques are not considered very reliable and may need more validation as well as standardization [37,38]. In one study, T2 magnetic resonance-based diagnosis was found effective for the diagnosis of *Candida* species [39,40]. Recently, a hyperspectral confocal fluorescence microscopy-based method has been described for identifying different *Candida* species [41]. Although these detection techniques work well for the diagnosis of *C. albicans*, these techniques show less specificity and sensitivity to non-albicans *Candida* species [32,42,43]. Therefore, improvement of the speed and accuracy of clinic diagnosis for invasive candidiasis is an important issue that must be resolved.

Serological diagnosis is one of the focuses of current research for the development of a diagnostic assay for invasive candidiasis [44]. This diagnostic strategy employs a specific interaction between the target antigen and antibody to detect the presence of infection. In this context, protein antigens that are recognized by the host’s immune system and involved in the pathogenesis of the infection might serve as useful serological markers for the development of a diagnostic assay. Serologic tests for invasive candidiasis allow for a short turnaround time. Hence, efforts have been made over recent decades to establish a serological diagnostic assay to identify systemic candidiasis patients. Consideration of the patient’s antibody response provides another option for systemic candidiasis diagnosis. The specific antibody response to *Candida* proteins that is usually induced in both immunocompromised and immunocompetent patients by invasive *Candida* infections is helpful in diagnosis. The Secreted aspartyl proteinase 2 (Sap2) protein, which belongs to the Sap gene family, is a promising antigen for the diagnosis of invasive candidiasis. The Sap2 protein functions as a hydrolytic enzyme, exhibiting broad substrate specificity, and is expressed abundantly in the culture of *C. albicans* [45]. As such, it is considered to be a leading vaccine candidate among the virulence-associated factors of *C. albicans.* In one study, a monoclonal antibody CAP1 was found effective for the detection of the Sap antigen, which indicated that the Sap-antigen-based ELISA assay could be used in the diagnosis of invasive candidiasis [46]. In another study, ELISA-based detection of the *Candida* Sap2 antigen has been demonstrated as an effective diagnostic for invasive candidiasis [47]. Although various detection assays are available for the diagnosis of invasive candidiasis, they are mostly non-specific and ineffective. Therefore, in the present study, we have used a recombinant Sap2 antigen from *C. parapsilosis* and evaluated its potential in the diagnosis of candidiasis. In this study, we report successful cloning, expression and purification of the recombinant Sap2 protein (rSap2) from *C. parapsilosis* in *E. coli*. The rSap2 protein was successfully refolded using a multi-step dialysis procedure and the secondary structure of refolded Sap2 protein was confirmed by CD and FTIR spectroscopy. Serological response against the rSap2 protein was investigated in mice using ELISA, demonstrating its immunogenic nature. In order to evaluate the diagnostic potential of the rSap2 protein during systemic candidiasis, the detection of anti-Sap2 antibodies in proven candidiasis patients and healthy controls was tested using immunoblotting and ELISA assays. Our results indicate that the rSap2 protein from *C. parapsilosis* can be used to detect and diagnose systemic candidiasis infection in human patients and can be used as an alternative/replacement of whole-cell *Candida*-based ELISA procedures, which are currently in use.

## 2. Materials and Methods

### 2.1. Chemicals and Strains

*C. tropicalis* (ATCC 750) and *C. parapsilosis* (ATCC 22019) were sourced from MTCC (IMTech, Chandigarh, India) and Dr. F. Khan (AMU, Aligarh, India) respectively. The pQE30Xa vector (Qiagen, Hilden, Germany) is used for the cloning and expression of the Sap2 protein with a 6-histidine tag at the N-terminus. Bacterial cultivations were performed in Luria-Bertani (LB) broth and agar with antibiotics (Ampicillin, 100 µg/mL and Kanamycin, 25 µg/mL). Enzymes for cloning and amplification were purchased from NEB. The *E. coli* XL1-blue and *E. coli* SG13009 strains were used for cloning and expression of the recombinant Sap2 protein. The Ni-NTA affinity matrix was purchased from Qiagen. Sabouraud’s Dextrose broth (SAB) and agar media were used for routine *Candida* culture experiments. Glycerol stocks of *C. parapsilosis* were used for inoculation and then cultivated at 30 °C in SAB broth for 48 h with continuous agitation.

### 2.2. Mice Information

Five- to six-week-old male inbred BALB/c mice were used in this study. All mice were purchased from IMTECH, Chandigarh and CDRI, Lucknow, India. Mice were maintained in the Institute Animal Facility under specific pathogen-free conditions at IIT Roorkee (Roorkee, Uttarakhand, India). All mouse experiments were performed using the protocols approved by the Institutional Animal Ethics Committee (IAEC) and according to committee recommendations and guidelines. Six mice per group were randomly distributed and housed in ventilated cages containing sterile bedding. Mice were fed sterile food pellets and water ad libitum. Prior to starting the mice experiments, mice were allowed to acclimatize to the new environment and entrained to a 12-h light/dark cycle at 25 °C.

### 2.3. Human Patients and Control Subjects

Human serum samples were obtained from PGIMER, Chandigarh, India, admitted between March 2018 to June 2018. The 20 subjects enrolled in this study were classified into two groups: Proven candidiasis patient group (*n* = 10) and control individuals (*n* = 10). For proven candidiasis subjects, the serum samples were obtained retrospectively within 24 h after an automated blood culture (BD BACTEC™ 9240, Franklin Lakes, NJ, USA) grew *Candida* species, which assured uniformity and that all subjects had an active disease at the time of enrollment. To provide data on assay specificity, the control individuals had similar age and sex distributions to the proven invasive candidiasis patients. The serum was separated, aliquoted and stored at 4 °C for up to 48 h or frozen at −80 °C until tested.

The study protocol was approved by the Ethics Committee of PGIMER and informed consent was obtained from all patients included in the study. All sera were analyzed in a blinded fashion. Data including age, primary condition and clinical stage were obtained from the clinical records. Baseline characteristics of the proven candidiasis patients are shown in Table 1.

### 2.4. Cloning and Expression of Recombinant Sap2 Protein from C. parapsilosis

The commercially available QIAamp DNA mini kit (Qiagen) was used for the genomic DNA isolation from *C. parapsilosis* according to the manufacturer’s protocol. PCR amplification of the Sap2 domain excluding the signal peptide region (referred to as the Sap2-sp fragment) was performed using specific primers and Phusion DNA polymerase (NEB). The 25 µL reaction mix contained 5X GC Buffer, 200 µM dNTP mix, 0.2 µM each of forward and reverse primers, 25 ng of *C. parapsilosis* genomic DNA and 2.5 Units of Phusion DNA polymerase. The PCR reaction was set up on a GeneAmp 2700 PCR system. PCR cycling conditions included initial denaturation at 98 °C for 2 min, followed by denaturation at 98 °C for 30 s, annealing at 56 °C for 1 min and an extension for 72 °C for 1.5 min, for a total of 30 cycles. A final extension step was carried out at 72 °C for 10 min. The *C. parapsilosis* Sap2-pp fragment was PCR amplified by the nested PCR strategy using *C. parapsilosis* Sap2-sp as a template and internal primers (Table 2). The composition of the reaction mixture and cycling parameters for the internal round PCR were the same as those used for the external round of PCR. The Sap2-pp fragment was purified and cloned into corresponding restriction sites of the *E. coli* expression vector pQE30Xa (Qiagen). The transformants were selected on LB ampicillin plates. Positive clones were identified by restriction digestion and confirmed by Sanger sequencing. For protein expression, the Sap2-pp construct was re-transformed in the *E. coli* strain SG13009 (Qiagen) using ampicillin and kanamycin as selection markers. The colonies obtained were processed with small-scale bacterial cultures. Protein expression was induced using isopropyl-β-D-thiogalactopyranoside (IPTG) at a final concentration of 1.0 mM for 8 h at 18 °C. The recombinant Sap2 protein was purified using Ni-NTA affinity chromatography following the manufacturer’s instructions (Qiagen) under denaturing conditions.

### 2.5. Refolding of Recombinant Sap2 Protein from C. parapsilosis

After protein purification with Ni-NTA chromatography, the histidine-tagged Sap2 fusion protein was confirmed on Western blots using the anti-His antibody (Thermo Scientific, Waltham, MA, USA). Protein concentrations were determined using the BCA method (Genetix Biotech, New Delhi, India). Thereafter, the refolding of denatured protein was optimized using a stepwise refolding (multi-step) dialysis procedure. In the stepwise refolding method, the purified Sap2 protein fractions were initially dialyzed against 6 M urea refolding buffer (containing 10 mM Tris pH 7.4, 100 mM NaCl, 1 mM DDT) at pH 7.4 for three hours at 21 °C. Subsequently, the Sap2-pp protein fractions were subjected to refolding steps in 4 M, 2 M and 1 M urea at 21 °C for a time duration of 3 h each. After 3 h, the protein fractions were dialyzed with gradually decreasing concentrations of urea (0.5 M, 0.25 M and 0.125 M) at 4 °C and a duration of 3 h each. The urea concentration was further gradually decreased to 0.0625 M, 0.03125 M and 0.0156 M concentrations, and protein fractions were dialyzed at 4 °C and a duration of 3 h each. Finally, the Sap2 protein fractions were subjected to dialysis in urea-free (0 M urea) dialysis buffer (10 mM Tris pH 7.4, 100 mM NaCl, 1 mM DTT and 2% glycerol) at pH 7.4 and 4 °C for 3 h with continuous stirring, to obtain the refolded Sap2 protein. Both the denatured and refolded protein fractions were used for subsequent structural analysis.

### 2.6. CD Spectroscopy

To evaluate the secondary structure content of the purified recombinant Sap2 protein, both the unfolded and refolded Sap2 proteins were subjected to CD analysis using a Chirascan Circular Dichroism Spectrometer (Applied Photophysics Ltd., Surrey, UK). CD spectra were collected using a 1 mm quartz cell under constant nitrogen purge between 190 to 250 nm in 1 nm wavelength steps with an average time of 2.0 s at 18 °C. Protein samples at concentrations of 1 mg/mL were analyzed in 10 mM Tris, pH 7.4 buffer containing 100 mM NaCl and 1M DTT. For each sample, five scans were collected and averaged. The final average spectrum of all the samples was estimated by subtracting the baseline spectrum of the corresponding buffer. The data were analyzed by the CAPITO web tool (https://capito.uni-jena.de, accessed on: 12 October 2021). Thereafter, the secondary structure-specific graphs were obtained by plotting the molar ellipticity against wavelength. Recorded CD data in millidegrees were converted into molar ellipticity as per the following formula: [Molar ellipticity (θ) = (m° × M)/(10 × L × C)], where θ (theta) is the molar ellipticity in deg.cm^2^.dmol^−1^, m° is the recorded ellipticity in millidegrees, M is the mean residual weight (g/mol), L is the path length in cm and C is the concentration in g/L [48]. Smoothening of the raw CD data was performed using the Savitzky–Golay filter in CAPITO software online using MAC OS version 11.1.

### 2.7. FTIR Spectroscopy

The secondary structure elements of the denatured and refolded Sap2 protein samples were further analyzed by Fourier transform infrared (FTIR) spectroscopy. A PerkinElmer Spectrum Two FTIR spectrometer was used to measure the spectrum in the spectral range of 400−4000 cm^−1^ at a resolution of 8 cm^−1^ and an average of 10 scans, using potassium bromide (KBr) pellets. FTIR spectroscopy was performed at the Department of Chemistry, IIT Roorkee.

### 2.8. Animal Immunization

After one week of adaptation, all mice (*n* = 6/per group) were subcutaneously immunized and boosted at days 0 and 21, respectively. The procedure used for the subcutaneous immunization of mice has been described earlier [49]. Briefly, 10 µg of purified rSap2-pp from *C. parapsilosis* was mixed with 50 µg of alum (Pierce) as the adjuvant in a volume of 100 µL under both the right and left hind thigh, using a 1-mL syringe fitted with a 26 G 1/2-gauge needle (BD, Singapore). Similarly, control mice were injected with 100 ul of a suspension of PBS and alum. Blood was collected retro-orbitally after immunization on days 7, 14, 21, 28, 35 and 42, and was allowed to clot at room temperature for 1 h. Mice sera were stored at −20 °C until they were used in ELISA.

### 2.9. Evaluation of Sap2-Specific Antibody Response in Mice

For determining Sap2-specific antibody titers, 96-well ELISA plates (Nunc MaxiSorp, Thermo Scientific, Waltham, MA, USA) were coated with 5 μg/mL of the recombinant Sap2-pp protein in carbonate-bicarbonate buffer overnight at 4 °C. All blocking steps were performed using 5% milk protein in PBS for 1 h at 37 °C and plates were washed with 0.05% Tween 20 in PBS (PBST) before use. The plates were then incubated with serially diluted serum samples obtained from mice immunized with rSap2-pp protein or sham-immunized controls. Serum samples were incubated for 1 h at 37 °C, following which the plates were washed, and Sap2-specific antibody levels were detected with HRP-conjugated goat anti-mouse IgG secondary antibody (Southern Biotechnology, Birmingham, AL, USA) (1:5000 dilution) for 1 h at 37 °C. Plates were subsequently developed with 0.4 mg/mL o-phenylenediamine dihydrochloride (SRL, Gurugram, India) dissolved in citrate-phosphate buffer (pH 5.0). The reaction was stopped with 2N H_2_SO_4_ and optical density at 490 nm was measured using a microplate reader (Tecan, Chapel Hill, Morrisville, NC, USA). Titers were defined as the highest dilution of serum that gave an optical density at least twice the mean background reading (wells with all reagents except sera).

### 2.10. Detection of Sap2-Antigens Using Human Serum Samples by Western Blot

The diagnostic potential of the rSap2 protein from *C. parapsilosis* was tested using a Western blot assay with candidiasis patient serum and healthy control serum samples. Briefly, 1 ug of the rSap2-parapsilosis protein was transferred to the nitrocellulose membrane (Bio-Rad, Hercules, CA, USA) and incubated with human serum samples diluted 1:100 in 1XTBST supplemented with 1% non-fat dry milk protein for 1 h at 37 °C with slow agitation. After three washes, the membranes were incubated with HRP-conjugated anti-human immunoglobulin G (IgG) secondary antibodies (Southern Biotechnology) diluted 1:3000 for 1 h at 37 °C. The subsequent procedure for Western blot was followed as described elsewhere.

### 2.11. Detection of Sap2-Specific and Candida-Specific Antibodies by ELISA in Human Serum

The serum samples of candidiasis patients (*n* = 10) were obtained from the Postgraduate Institute of Medical Education and Research (PGIMER), Chandigarh, India. Sera from ten healthy volunteers with no evidence of localized or systemic candidiasis were used as controls. They were stored at −20 °C until use. Preliminary human serum Ig, IgG and IgM ELISA assays were performed to optimize the concentration of both recombinant Sap2 from *C. parapsilosis* and heat-killed *C. tropicalis* antigens. To determine Sap2-specific antibodies titers, 96-well microtiter plates (Nunc) were coated with 5 µg/mL of rSap2-parapsilosis diluted in carbonate-bicarbonate buffer and incubated overnight at 4 °C. Before the blocking step, the antigen suspension was then discarded, and plates were washed with PBS-Tween 20 (PBST). Blocking was performed using 5% milk protein in PBS for 1 h at 43 °C followed by washing with PBST. Thereafter, the optimal dilutions of patients’ and healthy controls’ sera were added in duplicate wells and incubated for 1 h at 37 °C. After washing with PBST, the Sap2-specific antibodies level was detected with HRP-conjugated goat anti-human Ig, HRP-conjugated goat anti-human IgG and HRP-conjugated goat anti-human IgM secondary antibody (Southern Biotech) and were used at 1:5000 dilution each, respectively. These plates were incubated for 1 h at 37 °C followed by washing. Then plates were subsequently developed with 0.4 mg/mL o-phenylenediamine dihydrochloride (SRL, Gurugram, India) dissolved in citrate-phosphate buffer (pH 5.0). The reaction was stopped via the addition of 2N H_2_SO_4_ to each well. Finally, the optical density of each separate well was measured at 490 nm using a microplate reader (Tecan, Chapel Hill, Morrisville, NC, USA). Titers were calculated as the highest dilution of serum that gave an optical density at least twice the mean background reading (no serum). To determine *Candida*-specific antibody titers, a 96-well microtiter plate was coated with HKC cells (1 × 10^6^ cells/well) overnight at 37 °C. Blocking and later steps were followed as described above.

### 2.12. Statistical Analysis

All statistical analyses were performed using GraphPad Prism software version 6.0 (San Diego, CA, USA). Statistical differences between different groups were analyzed using the Mann–Whitney U test, and a *p*-value of < 0.05 was considered statistically significant.

## 3. Results

### 3.1. Cloning of Recombinant Sap2 Protein

**Figure 1:**
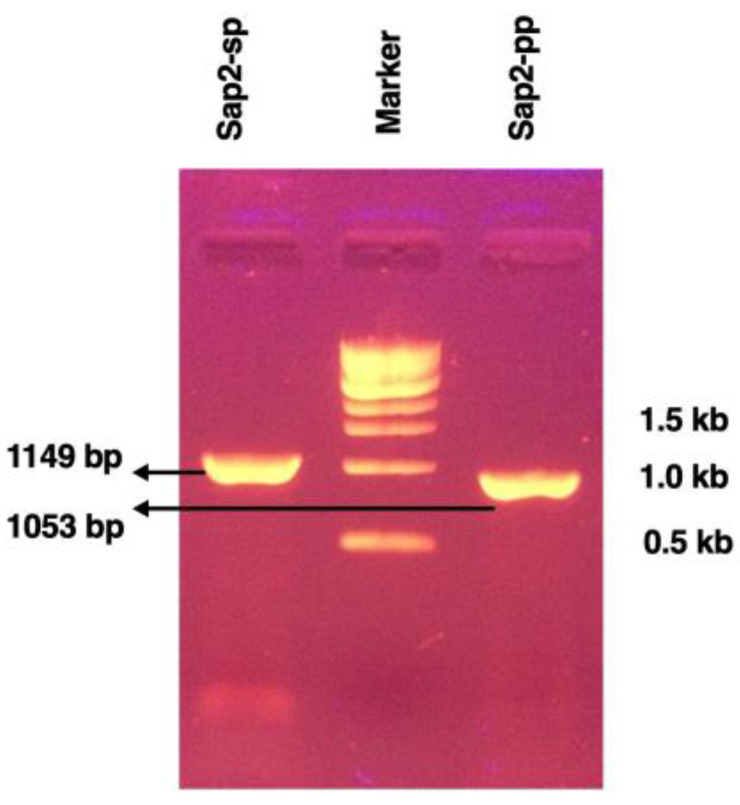
PCR amplification of Sap2 fragments from *C. parapsilosis* used in the study. PCR amplification of Sap2 gene excluding signal peptide (Sap2-sp: 1149 bp) and PCR amplification of Sap2 gene excluding both signal peptide and propeptide (Sap2-pp: 1053 bp). Marker: 1 kb DNA ladder (last three marker bands).

The Sap2 gene fragment was PCR amplified from genomic DNA of the *C. parapsilosis* strain (ATCC 22019, Genbank Z11918.1) using Sap2 gene-specific primers designed for the study (Table 2). Initially, the Sap2 gene excluding the signal peptide region (referred to as Sap2-sp) was obtained at 1149 bp, as indicated on agarose gel (Figure 1). Next, the Sap2-gene (excluding both signal peptide and pro-peptide regions; referred to as Sap2-pp) was PCR amplified using the Sap2-sp fragment as a template. A specific band of 1053 bp was obtained as shown in Figure 1.

The PCR amplicons were subjected to restriction enzyme digestions with Bam H1 and Hind III enzymes. After endonuclease digestion, the amplicons were cloned into the digested pQE30Xa vector with corresponding overhangs to enable directional cloning. The ligated products were transformed into *E. coli* XL-1 Blue strain competent cells. The colonies obtained after transformation were subjected to plasmid isolation and positive clones were identified by performing restriction analysis and confirmed by sequencing.

### 3.2. Expression and Purification of Recombinant Sap2 Protein

**Figure 2:**
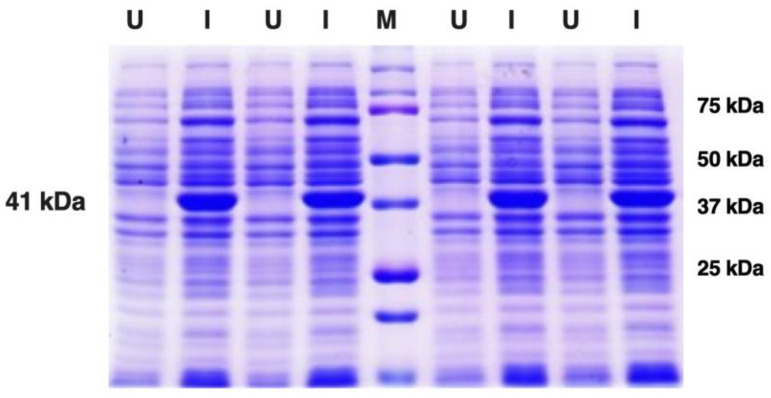
Expression of the rSap2-pp protein from *C. parapsilosis* used in the study. Induction of rSap2-pp protein: ~41 kDa; 62–412 aa. Lanes U: Uninduced samples; Lanes I: Induced samples; M: Protein Marker, Marker bands indicated on right.

For protein expression, the histidine-tagged fusion protein constructs of Sap2-pp were re-transformed in *E. coli* strain SG13009 (Qiagen). Small-scale *E. coli* bacterial cultures were subjected to IPTG induction with continuous agitation in an incubator shaker. Optimum protein expression was achieved at an IPTG concentration of 1 mM, a cell density (OD_600_) of 0.6 and an induction time of 8 h at 18 °C. The un-induced (U) and induced (I) bacterial cultures were analyzed using SDS-PAGE electrophoresis (Figure 2). A specific band corresponding to the molecular weight of approximately 41 kDa was obtained for the induced Sap2-pp protein, which was consistent with the expected size of Sap2 fusion protein.

**Figure 3:**
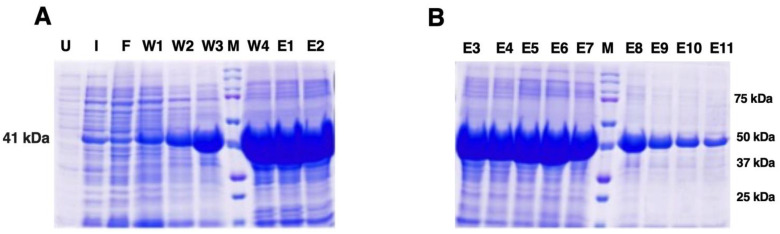
SDS-PAGE analysis of purified His-tagged rSap2 protein used in the study. Fractions of purified protein obtained under denaturing conditions (8.0 M Urea, 0.1% Triton X-114, pH 7.4) with different pH. (**A**,**B**) rSap2-pp protein (351 aa; ~41 kDa). Lane U: Uninduced sample; Lane I: Induced sample; Lane F: Flow through; Lanes W1-W4: Wash fractions; Lanes E1-E11: Eluted fractions; M: Protein Marker, Marker bands indicated on right.

The rSap2-pp protein was subsequently purified using Ni-NTA affinity chromatography following the manufacturer’s instructions (Qiagen) under denaturing conditions. SDS PAGE analysis shows fractions of the purified Sap2-pp protein obtained after washing and elution with buffers of different pH levels (Figure 3). The purified Sap2 protein (351 aa) was obtained at approximately 41 kDa.

### 3.3. In Vitro Refolding of Recombinant Sap2 Protein

**Figure 4:**
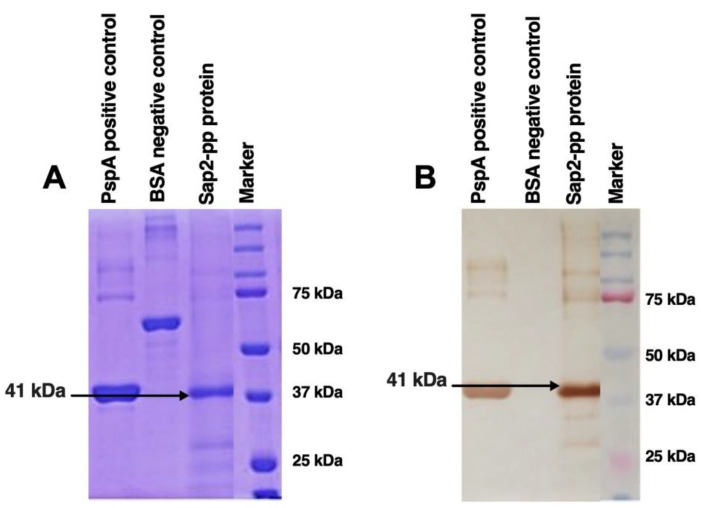
Western blotting of rSap2-pp protein used in the study. (**A**) SDS PAGE using Coomassie Brilliant Blue staining. (**B**) Western Blot using DAB substrate. Histidine tagged rSap2-pp protein obtained at ~41 kDa. PspA and BSA proteins are used as positive and negative controls, respectively. Protein Marker band sizes are indicated on right.

The purified Sap2 fusion protein (~41 kDa) was confirmed by Western blotting using the anti-His antibody and appropriate controls (Figure 4). Next, the purified rSap2-pp protein fractions were refolded using a multi-step dialysis procedure, as described in the methods. The protein fractions were initially dialyzed against the refolding buffer containing 6 M urea at pH 7.4 for 3 h at 21 °C. Subsequently, the protein fractions were dialyzed with gradually decreasing concentrations of urea (4 M, 2 M, 1 M, 0.5 M, 0.25 M, 0.125 M, 0.0625 M, 0.03125 M and 0.0156 M) at 21 °C and a duration of 3 h each. Finally, the rSap2-pp protein fractions were subjected to dialysis in urea-free (0 M urea) dialysis buffer at pH 7.4 and 4 °C for 3 h with continuous stirring to obtain the refolded rSap2-pp protein. Finally, the denatured and refolded protein fractions were processed with structural analysis.

### 3.4. Structural Analysis of Recombinant Sap2 Protein

**Figure 5:**
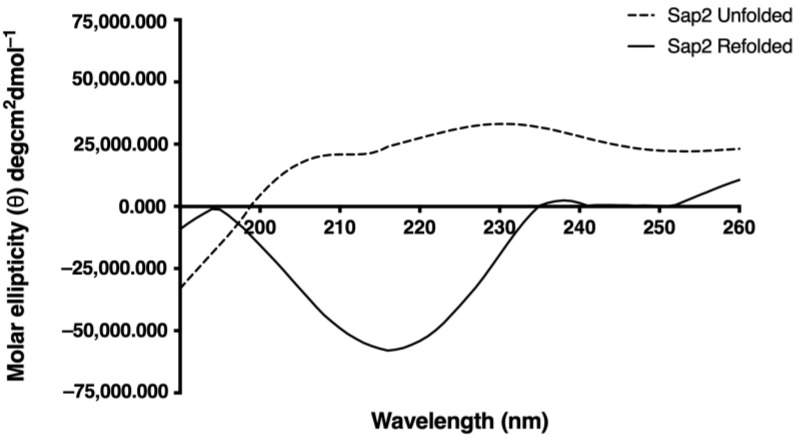
Far UV-CD spectra analysis of denatured and refolded rSap2-pp protein samples used in the study. CD spectra obtained for rSap2-pp protein is shown. Molar ellipticity is depicted on the y-axis with wavelength on x-axis. The denatured and refolded samples are shown in dotted lines and black lines, respectively.

We used CD spectroscopy to analyze the secondary structure of the denatured and refolded Sap2-pp protein. The CD spectral data were analyzed and deconvoluted using the CD spectra algorithm available online, as mentioned in the methods section. The CD spectra obtained for denatured and refolded forms of the Sap2-pp protein are shown (Figure 5). Molar ellipticity is depicted on the y-axis with the wavelength on the x-axis. Upon comparing CD spectra of the refolded and denatured Sap2-pp protein samples, a change in CD spectra was observed in the folded sample when compared with the denatured samples. As shown in Figure 5, a dip seen around 214 nm in the CD spectra for the refolded protein samples suggests the presence of secondary structure elements, especially β-strands [50], whereas the denatured samples depicted a predominantly random coil structure.

**Figure 6:**
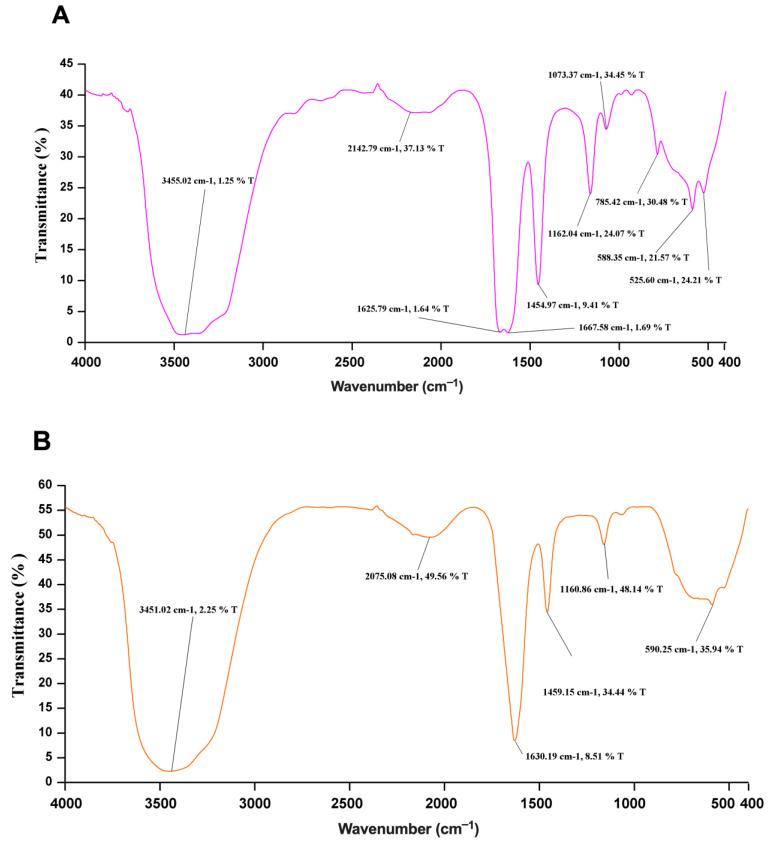
FTIR spectra of (**A**) denatured and (**B**) refolded rSap2-pp protein samples used in the study. (**A**) No characteristic peak obtained for the denatured rSap2-pp protein sample. (**B**) In the FTIR spectra, the sharp and prominent peaks obtained around at 1630 cm^−1^ indicate the presence of β-sheet rich structures in the refolded protein sample.

To further affirm the correctly folded state of the in vitro refolded Sap2-pp protein, the unfolded and refolded samples of the Sap2-pp protein were subjected to FTIR spectroscopy. The IR spectral data of proteins is usually interpreted in terms of vibrations of secondary structure repeat units, which give rise to nine absorption bands, namely amide A, B and I-VII. Among these, the amide-I band (1700–1600 cm^−1^), which is generated by the C = O stretch vibrations of the peptide linkages, is most useful in predicting the secondary structure of proteins. The amide bands in the regions of 1642–1624 cm^−1^ and 1696–1691 cm^−1^ correspond to β-sheets whereas amide bands in the regions of 1656–1651 cm^−1^ and 1685–1667 cm^−1^ corresponds to α-helix and β-turn secondary structures, respectively [50]. Compared to the denatured sample (Figure 6A), a sharp and distinct amide band was observed at 1630 cm^−1^ corresponding to β-sheet structures, in the case of the refolded Sap2-pp protein sample (Figure 6B). The spectral data also correlate with the crystal structures of the Sap2 protein from *C. parapsilosis* reported earlier [51]. In summary, the CD and FTIR data collectively suggest that the rSap2-pp protein attained its native conformation after refolding.

### 3.5. Evaluation of rSap2 Serological Response in Mice

**Figure 7:**
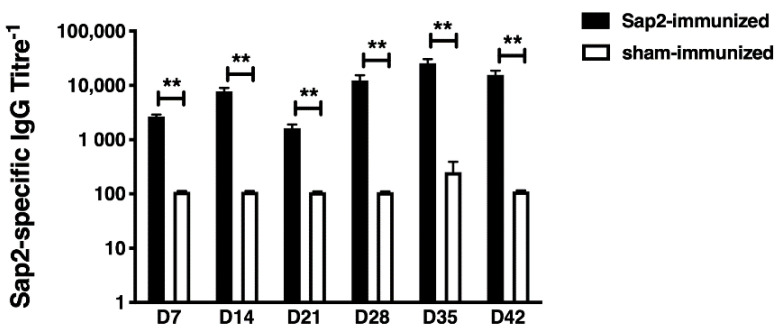
Serological response of Balb/c mice immunized with the rSap2-pp protein at different time points. Kinetics of rSap2-pp specific IgG antibody titers measured by ELISA on day 7, day 14, day 21, day 28, day 35 and day 42 in rSap2-pp immunized (black bars) and sham-immunized (open bars) mice. Antibody titers are shown on the y-axis and time in days is indicated on x-axis. All serum samples were tested in duplicate. Bars depict mean ± SEM (*n* = 6) and *p* values are calculated by the Mann–Whitney U test. Differences between groups are indicated by bars and symbols: ** *p* < 0.01.

In order to investigate the serological response of Balb/c mice against the rSap2-pp protein, the kinetic changes of IgG antibody titers to recombinant sap2 protein were evaluated at different time intervals. The rSap2-ppspecific IgG antibody titers were measured by ELISA assay on days 7, 14, 21, 28, 35 and 42 (Figure 7). Compared to sham-immunized mice (Av. titer 1:110), Sap2-specific total IgG antibody titers were significantly increased in rSap2-pp immunized mice (Av. titer 1:2680, *p* < 0.01) at day 7 post-immunization. The antigen-specific antibody titers further increased at day 14 in Sap2-immunized mice (Av. titer 1:7680) compared to sham-immunized mice (Av. titer 1:110, *p* < 0.01). After the booster dose at day 21, the Sap2-specific antibody titers increased gradually over time and reached their peak at day 28 (Av. titer 1:12,320) and day 35 (Av. titer 1:25,570), compared to sham-immunized mice at day 28 (Av. titer 1:110, *p* < 0.01) and day 35 (Av. titer 1:250, *p* < 0.01), respectively. The antigen-specific antibody titers gradually decreased in Sap2-immunized mice (Av. titer 1:15,640), but remained higher than sham-immunized mice (Av. titer 1:110, *p* < 0.01) at day 42 post-immunization. End point IgG titers of serum isolated from Sap2-immunized mice were highest 2 weeks after both initial and booster immunization (days 14 and 35). Based on the antibody titers, the recombinant Sap2-pp protein was found to be highly immunogenic in mice.

### 3.6. Diagnostic Potential of rSap2-Protein Using Immunoblotting

**Figure 8:**
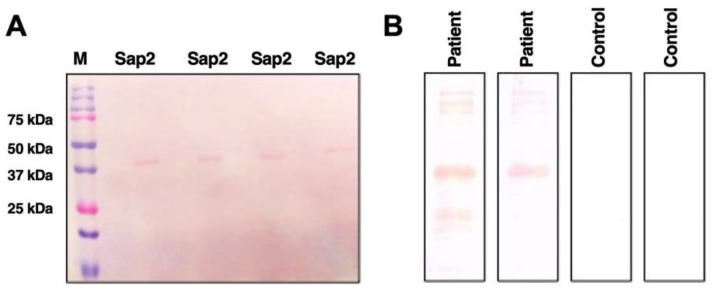
Recognition of rSap2-pp protein by candidiasis patient serum samples. (**A**) Western blot analysis using Ponceau S stain. Lane M: Protein marker (band sizes are indicated on left); Lane 1–4: Coated with Sap2-protein. (**B**) Western Blot analysis using DAB substrate. Lane 1–2: Candidiasis patient serum samples; Lane 3–4: Healthy human serum controls. Two strips are shown, representative of 10 samples, each group.

The reactivity of the rSap2 protein from *C. parapsilosis* for use as a potential diagnostic antigen was tested by immunoblotting with serum obtained from proven systemic candidiasis patients (*n* = 10) and normal healthy volunteers (*n* = 10). Western blot analysis demonstrated that the recombinant Sap2-pp protein was recognized as a single band of approximately 41 kDa by all ten systemic candidiasis patient serum samples (Figure 8). On the other hand, none of the serum samples obtained from the ten healthy volunteers exhibited any reactivity against the rSap2-pp protein. The rSap2-pp protein loading control is shown using Ponceau S stain in Figure 8A. The reactivity of the rSap2-pp protein with serum samples from two patients and from two healthy controls is depicted in Figure 8B (representative of all 10 samples). These results indicate that the detection of antibodies against the rSap2-pp protein by immunoblotting can be used as a specific and sensitive diagnostic test during systemic candidiasis.

### 3.7. Diagnostic Potential of rSap2-Protein Using ELISA

**Figure 9:**
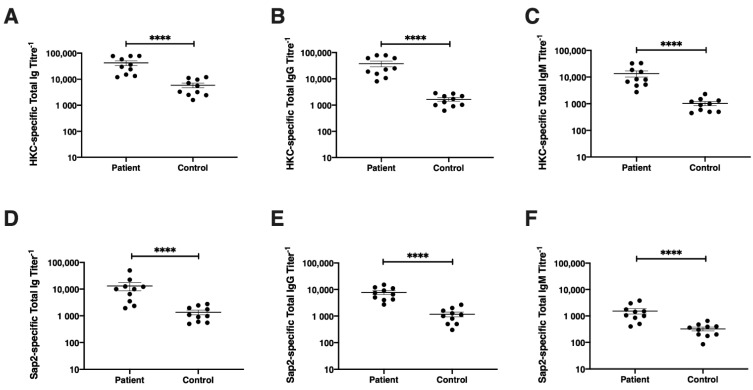
Detection of anti-*Candida* and anti-Sap2 Ig, IgG and IgM antibody titers in candidiasis patients. Serum *Candida*-specific total Ig (**A**), IgG (**B**) and IgM (**C**) antibody titers are shown for both patients and healthy controls. Serum-Sap2-specific total Ig (**D**), IgG (**E**) and IgM (**F**) antibody titers are shown for both patients and healthy controls. Antibody titers are shown on y-axis and serum samples are shown on x-axis (*n* = 10). Each black circle represents individual human serum sample. Graphs depict mean ± SEM and *p* values are calculated by Mann–Whitney U test. ****, *p* < 0.0001.

The diagnostic potential of the recombinant Sap2 protein during systemic candidiasis was further confirmed by *Candida*-specific and Sap2-specific ELISA assays. In order to determine the diagnostic accuracy of specific antibodies directed towards the immunodominant Sap2 antigen of *C. parapsilosis*, sera from candidiasis patients (*n* = 10) and healthy controls (*n* = 10) were investigated using ELISA (Figure 9).

Initially, proven systemic candidiasis patient serum samples were evaluated using whole *Candida*-specific total Ig, IgG and IgM ELISA. The anti-*Candida*-specific antibody titers in patients and healthy groups were evaluated using heat-killed *C. tropicalis* (HKC) as the coating antigen. HKC-specific Ig titers were found higher in candidiasis patient serum samples (Av. titer 1:42,220) compared to healthy controls (Av. titer 1:5860, *p* < 0.0001) (Figure 9A). Upon testing HKC-specific IgG titers, systemic candidiasis patients exhibited significantly higher titers of *Candida*-specific IgG antibodies (Av. titer 1:37,825), compared to healthy controls (Av. titer 1:1650, *p* < 0.0001) (Figure 9B). The HKC-specific IgM titers were also found to be significantly increased in patient serum samples (Av. titer 1:13,550) compared to healthy controls (Av. titer 1:1025, *p* < 0.0001) (Figure 9C).

Thereafter, the proven systemic candidiasis patient serum samples were tested by ELISA for the detection of Sap2-specific antibodies, along with serum from healthy individuals as controls. Upon evaluating Sap2-specific antibody titers, Sap2-specific total Ig titers were found to be significantly higher in proven candidiasis patients (Av. titer 1:13,150) compared to healthy controls (Av. titer 1:1350, *p* < 0.0001) (Figure 9D). Likewise, proven systemic candidiasis patients exhibited significantly higher Sap2-specific IgG antibody titers (Av. titer 1:7700) compared to healthy controls (Av. titer 1:1170, *p* < 0.0001) (Figure 9E). The Sap2-specific IgM titers were also significantly higher in candidiasis patients (Av. titer 1:1525) than healthy controls (Av. titer 1:325, *p* < 0.0001) (Figure 9F). Our results demonstrate that the rSap2 protein exhibited similar specificity and sensitivity in ELISA assays when compared to whole *Candida* antigen, confirming the utility of the detection of antibodies against the recombinant Sap2 protein for the diagnosis of systemic candidiasis. Taken together, our results indicate that thee rSap2 protein from *C. parapsilosis* can be used to detect and diagnose systemic candidiasis infection in human patients by ELISA and can be used as an alternative/replacement of whole-cell *Candida*-based ELISA procedures, which are currently in use.

## 4. Discussion

Since the signs and symptoms of invasive candidiasis are nonspecific, diagnosis remains a challenge. There are developing countries with a lack of available laboratory technology, where most *Candida* isolates are probably misdiagnosed. Because of a lack of rapid diagnostic assays for invasive candidiasis, most candidiasis cases are still diagnosed by routine fungal cultures of blood, urine tissue and other body fluids. *Candida* cultures and tissue histopathology from usually sterile sites remain the gold standard tests for the diagnosis of systemic candidiasis [52], which usually show low-reproducibility, low sensitivity and cross-reactivity problems. Microbiological evidence for the diagnosis of invasive candidiasis requires multiple, consecutive blood cultures. Previous studies demonstrated higher sensitivity of the CAGTA technique of invasive candidiasis, but this technique is difficult to automate and requires trained personnel to register microscopy results [53]. The diagnosis of invasive candidiasis is both clinically and microbiologically difficult [23]. To overcome this problem, diagnostic methods that are based on the detection of marker substances as well as antibodies to *Candida* in the serum have been used. Improved diagnostic tests for systemic candidiasis are among the most pressing needs in infectious diseases.

Despite numerous efforts and progress so far, exploration for an alternative diagnostic method is still ongoing. Importantly, the correct identification of *Candida* species is necessary to avoid inappropriate therapy. The detection of antibodies against different *Candida* antigens may help in the diagnosis. Hence, serological tests based on the detection of circulating antigens or anti-*Candida* antibodies from infected individuals are an attractive option for improved diagnosis of invasive candidiasis. Previous studies have shown that recombinant antigens have great potential as diagnostic reagents and support the utility of serology method for the diagnosis of invasive candidiasis [54,55,56,57]. Antigens from cell wall components such as mannan, galactomannan, and b-D-glucan have been widely studied for their diagnostic utility [30,58]. Sendid et al. reported *Candida* mannan and anti-mannan detection techniques for the diagnosis of systemic candidiasis, but they do not differentiate between *Candida* colonization and invasion [58]. Another cell wall component, b-D-glucan (BDG), has been used for the detection of *Candida* infection, which is associated with low specificity for invasive candidiasis [59]. The detection of antigens from the infecting agent in host samples is considered one of the practical strategies due to high specificity. Besides, several immunogenic recombinant proteins such as Sap2 (secreted aspartyl proteinase 2), Met6 (methionine synthase 6), Hsp90 (heat shock protein 90), Pgk (phosphoglycerate kinase), Eno1 (enolase 1), Hyr1 (hyphally regulated protein), Ece1 (extent of cell elongation protein 1), Als3 (agglutinin-like sequence 3), Bgl2 (1,3 β-glucanosyltransferase 2), Pdc11 (pyruvate decarboxylase 11), Fba1 (fructose bisphosphate aldolase 1), Adh1 (alcohol dehydrogenase 1), Grp2 (methylglyoxal reductase) and Hwp1 (Hyphal wall protein 1) have also been investigated for their potential as candidates for the diagnosis of invasive candidiasis [33,60,61,62], which could be useful as serum biomarkers [63]. Previously published studies demonstrated the development of both immunoblotting and an enzyme-linked immunosorbent assay (ELISA) for the serodiagnosis of invasive candidiasis by detecting specific antibodies against a recombinant N-terminal fragment of Hwp1 [62,64]. Laín et al. studied the reactivity of a recombinant Hyr1 protein with invasive candidiasis patients’ sera along with controls via immunoblotting [63]. Another study by Quanping et al. reported the Hsp90 antibody can be detected using a hybrid phage displaying thee antigen epitope in systemic candidiasis patients [65]. Among the recombinant antigen, enolase has been the most-studied antigen in the diagnostic utility of the detection of antibodies with invasive candidiasis patient sera [57,64,65,66,67,68].

In the current study, we report Sap2-specific immunoblotting and antigen-based ELISA techniques for the serodiagnosis of systemic candidiasis. Using a Sap2-specific ELISA assay, which detects specific Ig, IgG and IgM antibodies against the recombinant Sap2 protein, we evaluated the diagnostic potential of the Sap2 protein by comparing serum reactivity in candidiasis patients and healthy controls. Our results revealed that anti-Sap2 antibodies were found significantly higher in candidiasis patients than the control groups and can be used as an alternative to the whole-*Candida*-based ELISA assay (Figure 9). The Sap2 protein is a highly immunodominant antigen and virulence factor associated with fungal adherence, tissue invasion and dissemination in animal models of infection [49,69,70]. Sap2 proteinase contributes to host tissue damage [71,72], resists host antimicrobial activities by inhibiting complement [73] and Sap2 mutant *Candida* strains and exhibits reduced potential to cause damage [74,75]. The recombinant Sap2 protein can induce antibody production [49] and is a potential antigenic candidate for systemic candidiasis diagnosis. Previously, we reported the cloning, expression and purification of Sap2 protein from C. albicans and non-albicans *Candida* species using an *E. coli* expression system [49]. The advantage of using thee anti-Sap2 antibody as a serodiagnostic marker for candidiasis lies in its potential to differentiate between simple oropharyngeal and invasive candidiasis [76]. In this study, we amplified the Sap2 gene from the genomic DNA of *C. parapsilosis* using Sap2-gene-specific primers (Table 2). The Sap2 gene fragment lacking both a signal peptide and propeptide region (referred to as rSap2-pp) corresponding to 1053 bp was used in the study (Figure 1). The rSap2-pp protein was successfully expressed and purified in the bacterial expression system under denaturing conditions, using Ni-NTA chromatography. SDS PAGE analysis showed a distinct band corresponding to a molecular weight size of approximately 41 kDa, obtained for the rSap2 protein (Figure 3). The denatured protein was subjected to refolding using a multistep optimized refolding procedure. Structural analysis of refolded rSap2-pp using CD (Figure 5) and FTIR (Figure 6) confirmed that the refolded sSap2 protein was present in its near native conformation.

Reports have shown that Sap2 acts as a potential marker of candidiasis and can be utilized for its diagnostic purpose. Sap2 is known to appear in the early stages of *Candida* infection and diagnosis of candidiasis using anti-Sap2 monoclonal antibodies has been reported [46,76,77]. A study by Ghadjari et al. reported that anti-Sap2 antibodies are present in the recovered patients from systemic candidiasis [78]. Therefore, the detection of the Sap2 antibody also helps in both diagnosing the systemic candidiasis as well as in monitoring the response to anti-fungal treatment. A study by Na and Song demonstrated that inhibition ELISA with a monoclonal antibody (CAP1) is effective in the detection of thee circulating SAP antigen and that this assay may be useful for the diagnosis and treatment monitoring of invasive candidiasis [46]. Yang et al. demonstrated that a hybrid phage displaying a specific Sap epitope (VKYTS) show reactivity against serum obtained from *Candida*-infected mice and patients. By employing immunoblotting and ELISA-based techniques, they reported high specificity and sensitivity of Sap2-based immunoassays for the diagnosis of invasive candidiasis [76,79]. Quanping et al. evaluated the diagnostic potential of Hsp90 and Sap2 in systemic candidiasis and reported that anti-Sap2 antibodies were detected earlier than anti-Hsp90 antibodies in systemic infection, indicating its role in the early diagnosis of invasive candidiasis [65]. In a separate study, Wang et al. demonstrated that the detection of the Sap2 protein in serum samples of cancer patients using an ELISA assay may aid in the diagnosis of candidiasis [47]. The study further reported that using the Sap2 protein in ELISA-based diagnostics could be cost-effective, simple and rapid for the early detection of *Candida* infection.

In the present study, we evaluated the serological response in rSap2-pp immunized mice by analyzing the Sap2-specific antibody titers using ELISA over a period of time (Figure 7). The rSap2 protein was found to be highly immunogenic, similar to previously published reports [49]. Since conventional diagnostics methods for candidiasis show less sensitivity and specificity, novel immunodiagnostic techniques could therefore be explored for early diagnosis [80]. Our immunoblotting results demonstrate that the recombinant Sap2-pp protein was recognized as a single band of approximately 41 kDa by all ten systemic candidiasis patient serum samples and no cross reactivity was found with healthy controls (Figure 8). On evaluating the diagnostic potential of the rSap2 antigen using an ELISA-based approach, our results show that anti-Sap2 Ig, IgG and IgM antibodies could be detected in the sera of proven candidiasis patients. Of note, the differences in Sap2 antibody titers observed amongst patients and controls were similar to the serological response observed when whole heat-killed *Candida* was used as a coating antigen (Figure 9). Our results demonstrate that rSap2-protein-based ELISA can be developed as a rapid, convenient and cost-effective diagnostic test for systemic candidiasis as compared to conventional, time-consuming, heat-killed, *Candida*-based assays.

## 5. Conclusions

During the past few decades, the spectrum of *Candida* infections has changed drastically, and a shift from *C. albicans* to non-albicans *Candida* infections has been observed. Early diagnosis of systemic candidiasis is an important factor in decreasing patient mortality. There is an increasing interest in the development of new, reliable and simple diagnostic tests for the diagnosis of invasive candidiasis. In this study, we explored the potential of the recombinant Sap2 protein obtained from *C. parapsilosis* in the diagnosis of candidiasis by immunoblotting and ELISA assays. In this study, the Sap2 gene from *C. parapsilosis* was successfully cloned, expressed and purified using an *E. coli*-based prokaryotic expression system. We describe a detailed method for refolding the denatured recombinant Sap2 protein obtained from inclusion bodies. Our results from CD and FTIR spectroscopy confirm that the refolded rSap2 protein was obtained in its native conformation. The rSap2 protein was found to be highly immunogenic as evidenced from high titers of antigen-specific antibodies induced in rSap2-immunized mice. The diagnostic potential of the rSap2 protein was investigated using proven candidiasis patient samples and healthy controls. Both immunoblotting and ELISA results demonstrated specific reactivity of the purified recombinant Sap2 protein with patient serum. Our results suggest that measurement of the Sap2 specific antibody titers in candidiasis patient serum samples can contribute to rapid diagnosis of systemic candidiasis. The recombinant Sap2 protein has the potential to be used in the diagnosis of systemic candidiasis and could provide new ways for an accurate diagnosis of invasive candidiasis. Further, evidence of the immunological response of patients against the rSap2 protein can help in uncovering potential targets for vaccine design and immunotherapy against systemic candidiasis. In conclusion, the recombinant Sap2 protein from *C. parapsilosis* could be a promising candidate in the diagnosis as well as treatment of systemic candidiasis.

## Figures and Tables

**Table 1:** Baseline characteristics of human patients with candidiasis included in the study (*n* = 10).

S.N	CR No.	Age/Sex	Hospital Ward	Site of *Candida* Isolation	Isolate	Lab ID.
1	20180-2398021	72Y/F	General surgery/EMG Ward	Peripheral catheter blood	*Candida krusei*	3515
2	20170-6584839	64Y/F	General surgery/EMG Ward	Peripheral catheter blood	*Candida tropicalis*	40685
3	20170-6584840	60Y/M	General surgery/EMG Ward	Peripheral catheter blood	*Candida albicans*	40561
4	20180-1997525	53Y/M	Nephrology/Ward 22	Peripheral catheter blood	*Candida albicans*	40701
5	20180-1654784	79Y/M	Advanced Urology ward	Peripheral catheter blood	*Candida albicans*	40891
6	20180-1787504	17Y/M	Cardiology/Ward A	Peripheral catheter blood	*Candida albicans*	1105
7	20180-2525899	2Y/M	APC EMG Ward 2B	Peripheral catheter blood	*Candida albicans*	106
8	20160-3375341	41Y/M	Hepatology/MMW	Peripheral catheter blood	*Candida glabrata*	1731
9	20180-2693279	43Y/M	Gastroenterology/EMG Ward 5	Peripheral catheter blood	*Candida albicans*	5118
10	20180-284280	41Y/F	General surgery/EMG Ward	Peripheral catheter blood	*Candida albicans*	5957

**Table 2:** Primers and restriction enzymes used for PCR amplification and expression cloning of Sap2 fragments from *C. parapsilosis*.

Sap2-Regions	Sap2 Amplicons	Primer Sequence with Restriction Enzyme	Base Pairs	Amino Acids ^b^	Position
Sap2-sp	Sap2 sp FP	CCCCGGATCCGCTAAAAGAGATGATAACCCTG	1149 bp	383 aa	(30–412 aa)
	Sap2 sp RP	CCCCCAAGCTTTTGAATATAACGATTGTAAAAAAAGG			
Sap2-pp ^a^	Sap2 pp FP	CCCCGGATCCTCTTCTCCATCATCACCATTGTAC	1053 bp	351 aa	(62–412 aa)
	Sap2 pp RP	CCCCCAAGCTTTTGAATATAACGATTGTAAAAAAAGG			

^a^ Sap2-pp fragment was obtained using nested internal primers and Sap2-sp external fragment as template. ^b^ Besides the corresponding amino acids for Sap2-pp fragments (shown in bold) constructs included additional 31 aa from vector.

## Data Availability

Not applicable.
